# Biological adhesion of the flatworm *Macrostomum lignano* relies on a duo-gland system and is mediated by a cell type-specific intermediate filament protein

**DOI:** 10.1186/1742-9994-11-12

**Published:** 2014-02-12

**Authors:** Birgit Lengerer, Robert Pjeta, Julia Wunderer, Marcelo Rodrigues, Roberto Arbore, Lukas Schärer, Eugene Berezikov, Michael W Hess, Kristian Pfaller, Bernhard Egger, Sabrina Obwegeser, Willi Salvenmoser, Peter Ladurner

**Affiliations:** 1 Institute of Zoology and Center of Molecular Bioscience Innsbruck, University of Innsbruck, Technikerstr. 25, Innsbruck A-6020, Austria; 2 Evolutionary Biology, Zoological Institute, University of Basel, Vesalgasse 1, Basel CH-4051, Switzerland; 3 European Research Institute for the Biology of Ageing, University of Groningen, University Medical Center Groningen, A. Deusinglaan 1, Groningen NL-9713 AV, The Netherlands; 4 Division of Histology and Embryology, Medical University Innsbruck, Müllerstrasse 59, Innsbruck A-6020, Austria; 5 Department of Genetics, Evolution and Environment, University College London, Gower St, London WC1E 6BT, UK

**Keywords:** Flatworm, Intermediate filaments, Duo-gland system, Attachment, Bioadhesion

## Abstract

**Background:**

Free-living flatworms, in both marine and freshwater environments, are able to adhere to and release from a substrate several times within a second. This reversible adhesion relies on adhesive organs comprised of three cell types: an adhesive gland cell, a releasing gland cell, and an anchor cell, which is a modified epidermal cell responsible for structural support. However, nothing is currently known about the molecules that are involved in this adhesion process.

**Results:**

In this study we present the detailed morphology of the adhesive organs of the free-living marine flatworm *Macrostomum lignano*. About 130 adhesive organs are located in a horse-shoe-shaped arc along the ventral side of the tail plate. Each organ consists of exactly three cells, an adhesive gland cell, a releasing gland cell, and an anchor cell. The necks of the two gland cells penetrate the anchor cell through a common pore. Modified microvilli of the anchor cell form a collar surrounding the necks of the adhesive- and releasing glands, jointly forming the papilla, the outer visible part of the adhesive organs. Next, we identified an intermediate filament (IF) gene, *macif1*, which is expressed in the anchor cells. RNA interference mediated knock-down resulted in the first experimentally induced non-adhesion phenotype in any marine animal. Specifically, the absence of intermediate filaments in the anchor cells led to papillae with open tips, a reduction of the cytoskeleton network, a decline in hemidesmosomal connections, and to shortened microvilli containing less actin.

**Conclusion:**

Our findings reveal an elaborate biological adhesion system in a free-living flatworm, which permits impressively rapid temporary adhesion-release performance in the marine environment. We demonstrate that the structural integrity of the supportive cell, the anchor cell, is essential for this adhesion process: the knock-down of the anchor cell-specific intermediate filament gene resulted in the inability of the animals to adhere. The RNAi mediated changes of the anchor cell morphology are comparable to situations observed in human gut epithelia. Therefore, our current findings and future investigations using this powerful flatworm model system might contribute to a better understanding of the function of intermediate filaments and their associated human diseases.

## Background

Biological adhesion is a prerequisite for many organisms to accomplish critical tasks of life, and a broad range of organisms are able to attach to a variety of different surfaces, even under extreme environmental conditions [1-3]. For example, geckos are well-known for their impressive climbing capabilities relying on millions of small hair-like structures [4-7]. In contrast, aquatic organisms such as blue mussels, acorn barnacles, sandcastle worms, starfish, the freshwater caddisfly, and flatworms secrete adhesives to attain permanent or temporary attachment. The blue mussel *Mytilus edulis* attaches to the substrate using an apparatus called the byssus. It is composed of bundles of threads that terminate in the byssal plaque, which attaches to the substrate. The molecules of the plaque have already been identified [8-13]. Substantial progress has been made in characterizing the cement glue of barnacles [14-18]. The barnacle cement glands are huge cells secreting a proteinaceous substance containing more than 10 proteins into a duct, which is then secreted as the cement, a self-organizing, multi-functional complex that serves to permanently attach the animals to the substrate [18]. Adhesive secretions are also produced by the disc of the tube feet of echinoderms, which adhere and release from the substrate by means of a duo-gland system [19,20]. The composition of the involved proteins and the carbohydrate components has recently been analyzed for the sea star *Asterias rubens*[21,22] and the sea urchin *Paracentrotus lividus*[23,24]*.* Lectin staining has also been applied in planarian flatworms to label subepidermal marginal adhesive gland cells [25].

The glue of the sandcastle worm has been analyzed in detail [26]. Two secretory cells expel vesicles at the building organ, i.e. the structure used to assemble a tube-shaped housing consisting mostly of sand granules. One secretory cell contains homogeneous granules with polycationic Pc2 and Pc5 proteins, and the second secretory cell holds heterogeneous granules with oppositely charged polyphosphoproteins Pc3A/B and the polybasic proteins, Pc1 and Pc4. Together with additional components the vesicles are secreted and the mixture cures within 30 seconds to form the glue [27]. The caddisfly larvae spin adhesive silk to capture food and to construct a cover for protection and camouflage. Caddisfly silk fibers are composed of heavy- and light-chain fibroin protein linked by disulfide bridges [28-30]. The exact mechanism how silks stick underwater is not yet understood. Most likely phosphorylated serines and the presence of surface exposed phosphates play a role in underwater adhesion [31]. Parasitic Platyhelminthes use specialized morphological adaptations and adhesive secretions to adhere to their respective host [32]. For free-living flatworms the morphology of adhesive organs of a broad range of flatworm species has been analyzed [33-37]. A duo-gland adhesive and release system has been proposed [33,38]. Each duo-gland organ consists of at least three cells: One or more adhesive gland cells with electron-dense granules form the adhesive, and one or more releasing gland cells possessing smaller, less dense granules. These gland cells expel their secretions through a modified epidermal cell, called the anchor cell. Several lines of evidence support the suggestion concerning the function of the respective gland cell type [33]. The notion that the large dense granules of the adhesive cells are responsible for adhesion relies on observations of animals that were fixed during the adhesive process in the rhabdocoel flatworm *Messoplana falcata*, where the two gland cell types emerge in spatially separate papillae. Only adhesive gland cell necks were surrounded by a distinct microvilli collar while releasing gland necks were devoid of such a tension mediating structure (the same observation was also made in the polyclad *Theama sp.*). It was evident that secreted material was only found in vicinity of the adhesive gland tips. Furthermore, adhesive papillae of animals that were fixed during adhesion exhibited signs of tension. These papillae were bent in oblique angles (due to pulling forces) with respect to the epidermal surface and they were additionally stretched outwards. This was never observed for releasing gland papillae and adhesive papillae that did not participate in this adhesion incident (see [35] for details). According to the conserved nature of the structural components of the adhesive organs we assume that the cell containing the large dense granules represents the adhesive cell. Furthermore, in an undescribed planarian flatworm studied by Tyler “1976” at least 60 adhesive gland cell necks and more than 100 releasing gland cell necks penetrate a single anchor cell. Only adhesive gland cell necks are surrounded by a collar of microvilli corroborating the assumption that the gland cell with the dense granules is responsible for adhesion [33]. Tyler suggested tonofilaments in the cytoplasm of the anchor cells to direct the forces from the microvilli collar to the extracellular matrix. In the planarian *Dugesia japonica* Tazaki et al. (2002) [39] identified the intermediate filament *DjIFb* expressed in the epidermal layer of the adhesive organs [39]. Their observations pointed to an important role of intermediate filaments (IFs) in the adhesion process. IFs are essential structural elements of metazoan cells. They form resilient cytoplasmic and nuclear networks, providing mechanical strength to cells [40-42]. Their tight connection with desmosomes and hemidesmosomes dynamically anchors cells within the tissue. In contrast to microtubule and actin filaments, the expression of IFs is often cell-type or tissue specific. Therefore, IFs form a huge gene family and approximately 30 human diseases are related to mutations in these genes [43]. This high number reflects the importance of IFs in providing tissue function and integrity. In this study we use the flatworm *M. lignano* to analyze IF function during adhesion.

*Macrostomum lignano* is primarily used as a model in developmental and evolutionary studies [44-49]. It is small in size (up to 1.5 mm), highly transparent, can easily be cultured under laboratory conditions, and exhibits a comparatively simple organization of tissues and organs. It is an obligatorily cross-fertilizing hermaphrodite that produces eggs throughout the whole year in laboratory cultures. A broad methodological toolbox is available to study developmental processes, including *in situ* hybridization and RNA interference [50-53], cell- and tissue-specific monoclonal antibodies [54] and EST sequencing [55] (http://flatworm.uibk.ac.at/macest/). Both a preliminary genome and transcriptome are available to the public (http://www.macgenome.org/). *M. lignano* is a well-suited model system to study the distribution, differentiation, and migration of stem cells [56-61] as well as the expression and function of stem cell and germ line genes in adult animals, during postembryonic development and during regeneration [50,52]. *M. lignano* has a high regeneration capacity [62,63] and detailed studies on the regeneration of the region anterior to the eyes [61], head regeneration [49], and tail-plate regeneration [64] have been performed. In the present study we take advantage of the fact that *M. lignano* is able to completely regenerate an amputated tail-plate within less than 10 days [62,64].

Here we present the morphology of the *M. lignano* adhesive organs using light microscopy, scanning- and transmission electron microscopy, and phalloidin staining. We confirm that the *M. lignano* adhesive system consists of three cell types, an anchor cell, an adhesive cell, and a releasing cell. We next identified an intermediate filament gene that is essential for the proper function of the anchor cells. RNAi mediated knock-down of the respective intermediate filament mRNA resulted in a non-adhesive phenotype and corresponding morphological changes of the anchor cell. In summary, we present a detailed analysis of the morphology of the *M. lignano* adhesive system and a functional analysis of a gene found to be involved in the adhesion process.

## Results

### Morphology of the *Macrostomum lignano* adhesive system

**Figure 1:**
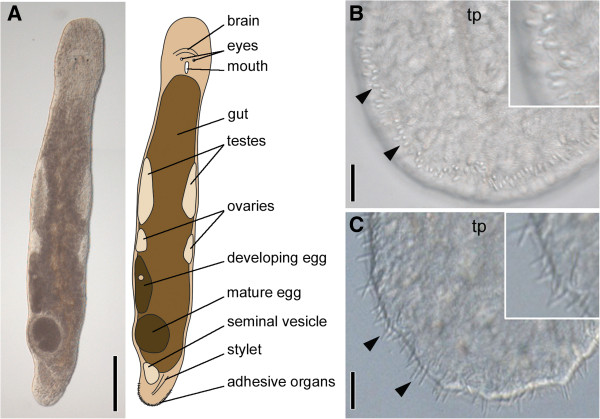
**Overview of the morphology of *Macrostomum lignano*. (A)** Interference contrast image and schematic drawing. **(B)** Unattached tail plate (tp) with relaxed adhesive organs (arrowheads). **(C)** Tail plate with adhesive organs attached to the glass slide (arrowheads). Scale bars 200 μm **(A)**, 10 μm **(B,C)**.

**Figure 2:**
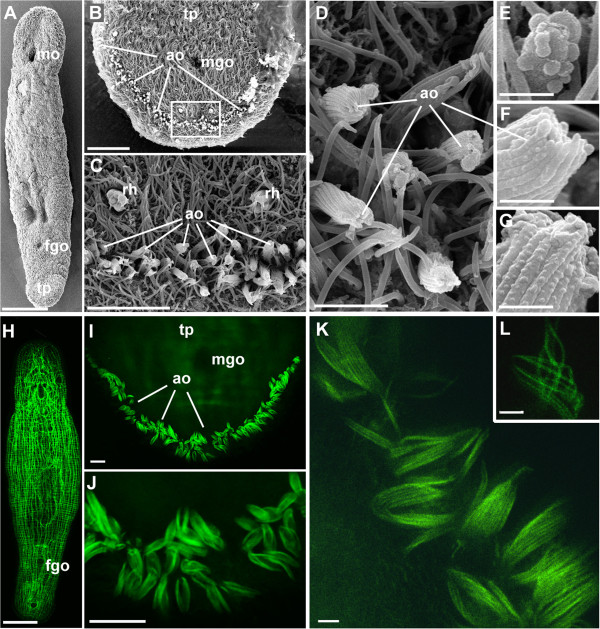
**Adhesive organs of *M. lignano *shown with scanning electron microscopy (A-G) and phalloidin staining of actin filaments (H-N).** Scanning electron microscopy overview **(A)**, tail-plate **(B)** and detail thereof **(C)**, and details of adhesive organs **(D-G)**. Note microvilli collar on the anchor cells **(D, F, G)** and adhesive vesicle on the tip of an adhesive organ **(E)**. Phalloidin staining overview **(H)**, tail plate **(I)** and details thereof **(J)**, and details of the adhesive papillae **(K, L)**. ao adhesive organ; fgo female genital opening; mgo male genital opening; mo mouth opening; tp tail-plate; rh rhabdites. Scale bars **(A, H)** 100 μm, **(B)** 20 μm, **(C, I, J)** 10 μm, **(D)** 2 μm, **(E-G)** 0.5 μm, **(K, L)** 1 μm.

The natural habitat of *Macrostomum lignano* (Figure 1A) is the sediment of sheltered beaches of the Northern Adriatic and possibly other sites in the Eastern Mediterranean [48]. Animals can be found within the oxygenized surface sand at the high-tide level. Like many other members of the meiofauna including other flatworms *M. lignano* has evolved mechanisms to maintain contact with the sand substrate against the action of water and tidal changes - namely an adhesive system, here positioned at the tip of the tail plate (Figures 1 and 2). In culture, *M. lignano* are kept in petri dishes where they glide along the bottom by ciliary gliding. The animals are able to adhere and release several times within a minute. Using interference contrast microscopy of squeezed live animals one can identify the external part of the adhesive organs, described as adhesive papillae [35], which are arranged horseshoe-like along the ventral side at the tip of the tail plate (Figure 1B). When the animals adhere to the surface the adhesive organs are slightly stretched above the epithelial surface (Figure 1C). In video investigations we observed that only a few adhesive organs were used for one adhesion incident (Lengerer, pers. observation).

**Figure 3:**
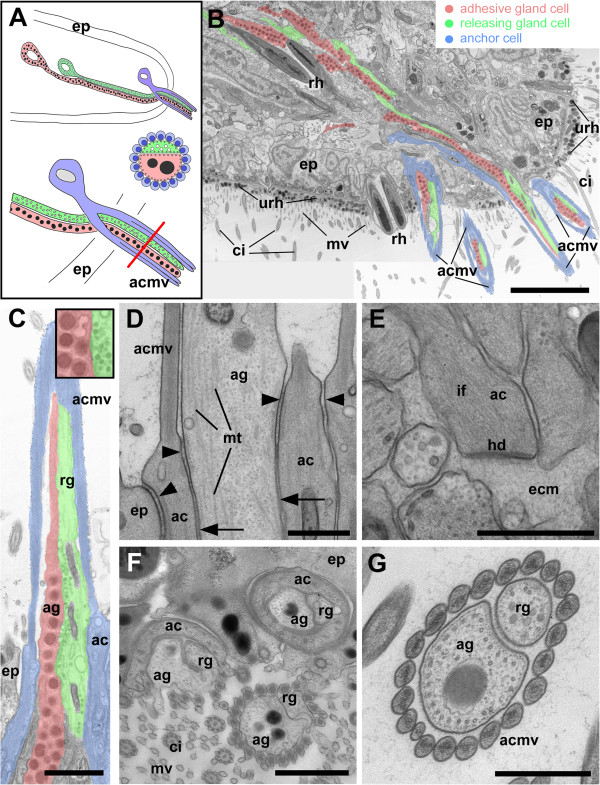
**Overview and fine structure of the *M. lignano *adhesive organs.** Schematic illustration **(A)** and transmission electron microscopy (cryo-processed specimens) **(B-G)**. **(A)** Localization of adhesive organ cell types: anchor cell (blue); adhesive gland (red); releasing gland (green). The red line indicates the level of the cross section of the adhesive papilla. **(B)** Sagittal section of the tip of the *M. lignano* tail plate. Four adhesive organs are visible and several gland cell necks reach into the tail plate. **(C)** Sagittal section of an adhesive organ. Inset: detail of respective vesicle types. **(D)** Section showing strengthened adherens junctions (arrowheads) and septate junctions (arrows) between an epidermal cell and an anchor cell and between an adhesive cell and an anchor cell. Note: microtubules (mt) within the adhesive gland. **(E)** Basal cytoplasmic extension of an anchor cell (ac) with intermediate filaments (if); the cell is connected to the extracellular matrix (ecm) via a hemidesmosome (hd). **(F)** Horizontal section through adhesive organs and an epidermal cell (ep) with cilia (ci) and microvilli (mv) protruding from the epidermal surface. Three adhesive organs are sectioned at different levels. Note that the anchor cell (ac) surrounds the adhesive gland (ag) and releasing gland (rg) cells in a donut-shaped manner, i.e. without cytoplasmic interruption. **(G)** Cross section through an adhesive organ with central adhesive gland (ag) and releasing gland (rg) cells surrounded by a collar of microvilli of the anchor cell (acmv). ac anchor cell; acmv anchor cell microvilli; ag adhesive gland; ci cilium; ecm extracellular matrix; ep epidermis; hd hemidesmosome; if intermediate filaments; mt microtubules; mv microvilli of regular epidermal cells; rg releasing gland; rh rhabdite glands, ultrarhabdites (urh). Scale bars **(A)** 5 μm, **(C,F)** 1 μm, **(D,E,G)** 0.5 μm.

About 130 adhesive organs are present in adult *M. lignano* (see also [62]). Scanning electron microscopy revealed that each organ consists of an array of dense microvilli (Figure 2). A view onto the tip of the papillae showed the ring-like arrangement of the distal-most tips of the microvilli collar, which was closed above the tips of the adhesive and releasing glands (Figure 2D-G). Occasionally, small droplets of secreted material can be seen on the tip of an adhesive organ (Figure 2E). The microvilli were composed of bundles of actin filaments and were visualized with phalloidin staining and confocal (Figure 2H-J) and superresolution microscopy (STED) (Figure 2K, L). Lateral views on the papillae revealed labelling of individual microvilli (Figure 2K, L). In sagittal TEM sections of adhesive organs their internal organization became obvious. Each adhesive organ was comprised of three cell types, i.e. one adhesive gland cell - also referred to as the viscid gland cell [33], one releasing gland cell, and one anchor cell (Figure 3). The anchor cell is a modified epithelial cell with long microvilli that were closely attached next to each other forming a palisade-like envelope (Figure 3A, F, G) for the necks of the adhesive and releasing gland cells. The microvilli of the anchor cells protruded from the epidermis and surrounded the distal-most tips of both the adhesive- and releasing gland cells (Figure 3A-C, G). Within the microvilli collar of the anchor cell the adhesive gland cell was always located at the ventral side, and the releasing gland cell at the dorsal side, respectively (Figure 3A, B; see also Additional file 1). The anchor cell lacked cilia, ultrarhabdites (epitheliosomes), and a terminal web (Figure 3B). The cell body of the anchor cell lay in the parenchyma below the body wall musculature. The necks of the adhesive- and releasing gland cells penetrated the anchor cell and emerged through the anchor cell body forming - surrounded by the microvilli collar - the adhesive papillae on the body surface. The cell bodies of the adhesive gland cells (Additional file 2A) lay further anterior in the tail plate at the level of the stylet and the prostatic glands. The cell bodies of the releasing gland cells (Additional file 2B) were located posterior to the adhesive gland cell bodies, although there was a region of overlap (Figure 3A, Additional file 2C). In serial TEM sections of one specimen no cell bodies of releasing- or adhesive gland cells were present up to 16 μm from the tip of the tail. Between 17 to 23 μm from the tip of the tail only releasing gland cell bodies were found. In the region from 24 μm to 37 μm both, releasing- and adhesive gland cell bodies were present. From 37 μm onwards to 83 μm from the tip of the tail only adhesive gland cell bodies existed.

At the ultrastructural level the adhesive cells possessed electron-dense spherical-ovoid secretion granules of about 270 nm in diameter (Figure 3C, Additional file 2A). The releasing cell granules were less dense and up to 70 nm in diameter (Figure 3C, Additional file 2B). The adhesive cell was attached to the anchor cell by a belt of apical adherens junctions (Figure 3D). Likewise the anchor cell was connected to the neighbouring epidermal cell by a strong junctional complex (Figure 3D). Both, the adhesive- and the releasing cell contained a microtubule system which was probably involved in vesicle transport (Figure 3D). In the anchor cell, tonofilaments were connected to the extracellular matrix via multiple hemidesmosomes (Figure 3E). The anchor cells formed a cylinder-shaped gapless tube of cytoplasm which appeared donut-shaped in TEM cross sections and was penetrated by the adhesive- and the releasing cell necks (Figure 3F). In the microvilli of the anchor cell, tightly bundled actin filaments were present and formed a dense core in the centre of the microvilli (Figure 3G). When the gland cell necks emerged at the surface of the anchor cell they were surrounded by 20–24 microvilli, forming together the adhesive papillae (Figure 3F, G). Overall, the adhesive organs comprise an elaborate structural interaction of three cell types and altogether the roughly 130 adhesive organs constituted the versatile adhesive system of *M. lignano.*

### An anchor cell-specific intermediate filament mediates adhesion of *M. lignano*

**Figure 4:**
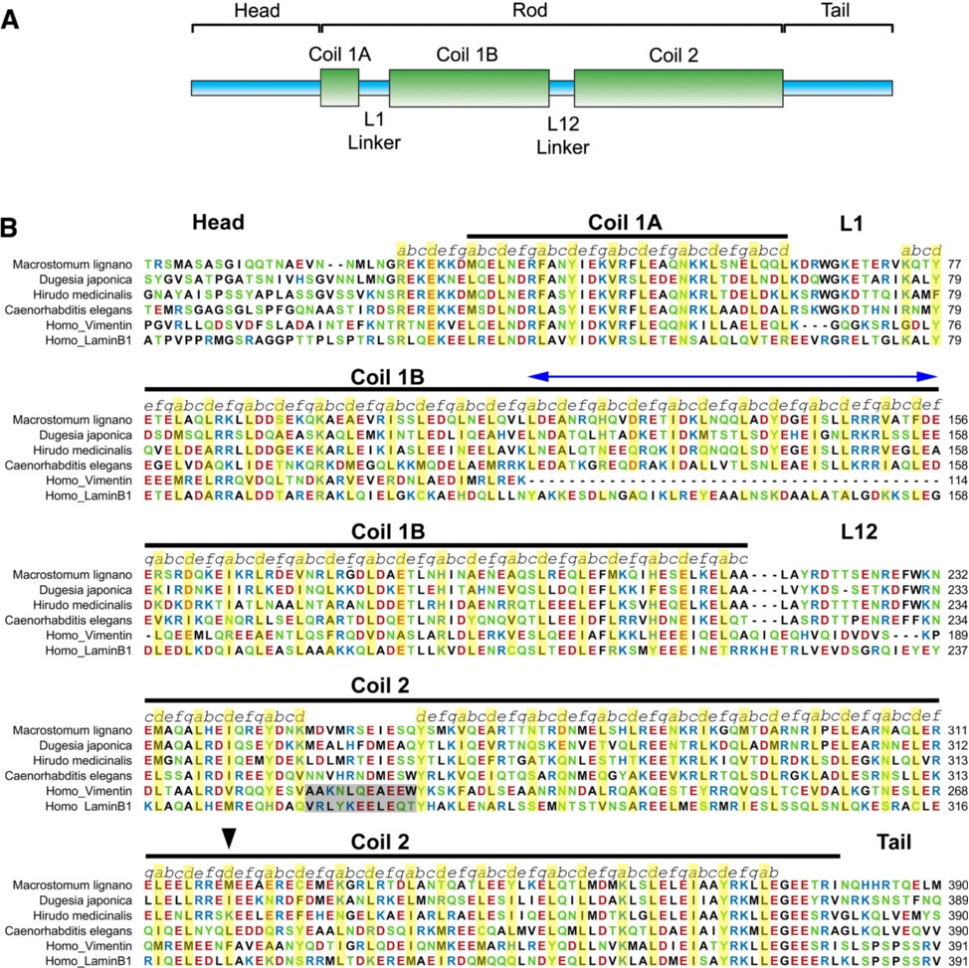
**Domain organization (A) and primary structure (B) of the Macif1 protein.** Hydrophobic residues are shown in black, hydrophilic residues in green, acidic residues in red and basic residues in blue. The characteristic heptad repeat pattern is shown as *“abcdefg”* and the apolar residues located at position “*a*” and “*d*” are indicated in yellow. The linker L2 that separates the coils 2A and 2B in vertebrate intermediate filaments is highlighted in grey. See text for more details.

We have identified an intermediate filament gene *macif1* (Figure 4) in an *in situ* hybridization screen [65] of a posterior-end specific transcriptome of *M. lignano*[66]. Details will be provided in future publications. Briefly, 200 animals were amputated posterior to the ovaries and 100 intact animals were used as control. For both samples 20 million Illumina reads (36 bp) were generated. The obtained reads were then mapped to the *M. lignano* transcriptome. In this way transcripts expressed in the posterior end were identified. From this dataset the expression of 48 genes was analysed and one was localized in the anchor cells of the adhesive organs. A BLAST search revealed an intermediate filament-like gene which we refer to as *macif1*. We cloned and sequenced *macif1* and identified an open reading frame of 1815 bp encoding for 605 amino acids. The primary amino acid sequence of Macif1 contained all domains characteristic for a bona fide invertebrate intermediate filament protein including a head-, a rod- and a tail domain (Figure 4A). Within the central rod domains a distinct periodic heptamer signature (indicated as”*abcdefg*” in Figure 4B) with a characteristic [67] distribution of apolar residues at the positions “*a*” and “*d*” (indicated yellow in Figure 4B) was present. In the central part of the coil 2 subdomain the heptad repeats were interrupted by a discontinuity, the so called stutter (indicated by an arrow in Figure 4B). The Macif1 predicted protein shared the long version of the 1B subdomain with six additional heptamers (indicated with a blue double headed arrow in Figure 4B) present in all protostome intermediate filaments and in vertebrate lamins, but not in e.g. human vimentin and other vertebrate intermediate filament proteins (Figure 4B). Two regions across all intermediate filaments are particularly well conserved. These regions play a role in dimer-dimer formation [68] and were also present in the predicted Macif1 protein. They span the first part of coil 1A and the very end of coil 2B. In summary, the structural organization of Macif1 confirms its close relationship to other invertebrate intermediate filament proteins.

**Figure 5:**
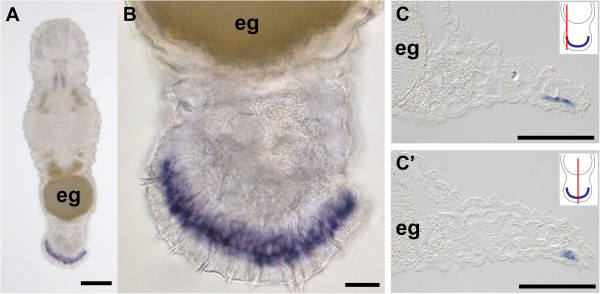
**Expression analysis of *macif1 *in *M. lignano.*** Whole mount *in situ* hybridization pattern **(A)** overview, **(B)** tail plate and **(C-C’)** semi-thin sections of the whole mount *in situ* hybridization. Schemes indicate the respective level of the semi-thin sections. (eg) egg. Scale bars **(A)** 100 μm, **(B)** 20 μm, **(C, D)** 50 μm.

**Figure 6:**
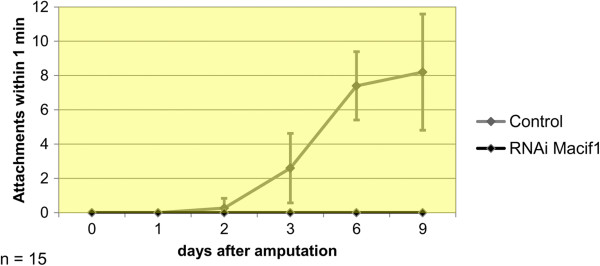
**Average number of attachments within one minute of *macif1 *dsRNA treated worms and controls.** Error bars indicate the standard deviation. On day 0 the tail plates were amputated and treatment with *macif1* dsRNA started. Controls were kept in f/2 medium. 1, 2, 3, 6 and 9 days post-amputation the counting of total attachments within one minute was performed for 15 individuals of the RNAi and control worms.

*Macif1* was expressed in a horseshoe-like belt along the margin of the tail plate (Figure 5A, B) corresponding to the location of the anchor cells (Figure 5C). Weak staining was also observed in the mouth region (see discussion). Functional analyses using RNA interference resulted in a non-adhesion phenotype. On days 1, 2, 3, 6 and 9 post-amputation 15 *macif1* dsRNA treated and control animals were individually observed for one minute to score their ability to adhere (Figure 6). RNAi treated animals move around in a petri dish but they are unable to perform a regular attachment. However, close observations revealed that animals performed an attachment movement by pressing the tip of the tail onto the surface. On these occasions a minimal delay of their forward movement was noticed. Since the adhesive gland cell was not affected by the RNAi treatment we hypothesize that animals secreted the glue and very briefly adhered to the surface. By no means this can be compared to a regular attachment process. No deformation of the tail plate can be observed which is significant during regular attachment (Additional file 3). Furthermore, the slightest movement of the petri dish or any minimal water current (Additional file 4) impeded any attachment of an RNAi treated animal. Under a binocular microscope the regular attachment can be very easily distinguished form the minuscule adherence of RNAi treated animals. While not a single individual of the *macif1* dsRNA treated animal was able to adhere effectively, the control animals did hold on to the slide up to 8.2 times per minute. These results corroborate the essential role of *macif1* for the *M. lignano* adhesion process.

We next investigated the ability of regenerating animals to recover from RNAi treatment. At day 13 post-amputation *macif1* dsRNA treated animals were not able to adhere to the substrate (Additional file 5). From day 14 post-amputation onwards these animals were transferred to normal culture medium and the recovery of adhesion was followed. After 24 days (11 days of recovery) 23% of the animals (three out of 13) were able to adhere, after 27 days (14 days of recovery) 46% (six out of 13), and after 30 days (17 days of recovery) 61% (8 out of 13), respectively, while 100% of the control animals (n = 9) showed multiple adhesion actions at any time point (Additional file 5). These findings indicate that recovery of the adhesive functionality after knock-down of *macif1* is slow compared to rapid regeneration of adhesion function after amputation.

**Figure 7:**
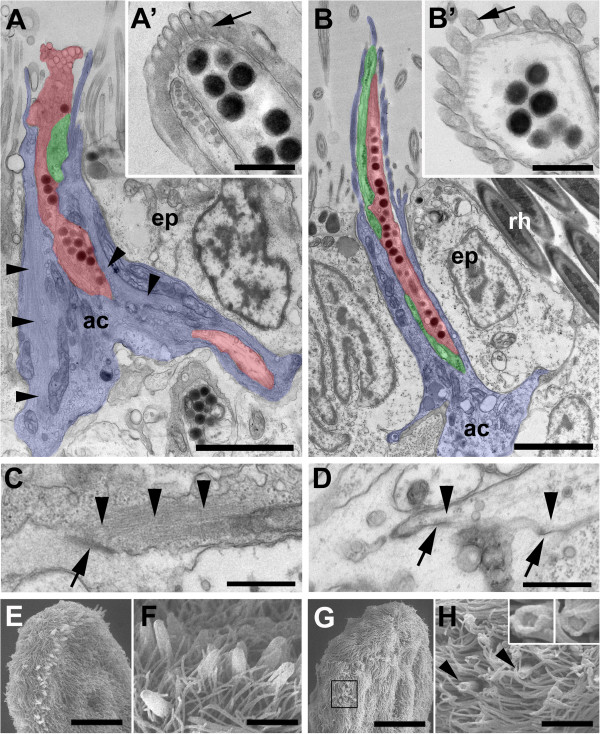
**Morphological comparison of controls and *macif1 *dsRNA treated animals at 9 days post amputation. (A-D)** TEM of a chemically fixed adhesive organ of a control **(A, C)** and a *macif1* RNAi treated animal **(B, D)**. Note that all three cell types are present in both treatment groups: anchor cell (blue), adhesive (red) and releasing gland (green). Arrowheads indicate intermediate filaments. Note the absence of intermediate filaments in the *macif1* RNAi treated specimen **(B)**. Insets **(A’, B’)** show a cross section of the adhesive organ outside of the epidermal layer. Note the different amount of actin filaments within the microvilli and the lack of the dense core in *macif1* RNAi treated animals (arrows). **(C, D)** Arrows indicate hemidesmosomes of a control **(C)** and a *macif1* RNAi treated animal **(D)**. Note the dense IF bundles connected to the hemidesmosomes in controls, are missing in *macif1* RNAi treated animals (arrowheads). **(E-H)** Scanning electron microscopy of adhesive organs at the tail plate and details. Note that the adhesive organs of control animals reach out above the cilia **(E, F)** while adhesive organs of *macif1* dsRNA treated animals are much shorter **(G, H)** and the microvilli core is open. Scale bars **(A, B, F, H)** 2 μm, **(A’, B’, C, D)** 0.5 μm, **(E, G)** 20 μm.

At nine days post-amputation a reduction of *macif1* mRNA in dsRNA treated animals was obvious (Additional file 6A, B). We next analysed the effect of *macif1* knock-down on the ultrastructure of the anchor cell. TEM revealed a drastic reduction of the size of the cytoplasm of the anchor cells and a lack of intermediate filaments (nine anchor cells from three different individuals) (Figure 7A-D). Not only the anchor cell bodies were affected, the absence of intermediate filaments also altered the structure and size of microvilli. The dense core of actin filaments in the microvilli was smaller or missing (arrow in Figure 7A’ , B’). This also became apparent with phalloidin staining and resulted in a reduced ability to label the anchor cell microvilli after six days of *macif1* RNAi treatment (Additional file 6C, D). Scanning electron microscopy corroborated the shortened microvilli in treated animals compared to controls (Figure 7E-H). In control animals the microvilli core appears closed (see also Figure 2D-G), while in treated specimen the opening is clearly visible (Figure 7H insets). These observations confirm the fundamental role of the cell type-specific intermediate filament *macif1* for *M. lignano* adhesion.

## Discussion

### Flatworm adhesive organs

A common feature for organisms living in a marine interstitial environment is their ability to adhere to the substrate in order to withstand water flow between sand grains. Indeed sandy beaches are regularly exposed to strong water currents as a result of tidal change and weather influences. Therefore, most members of the meiofauna, including cnidarians, nematodes, annelids, gastrotrichs, and free-living flatworms developed mechanisms to temporarily attach to surfaces. These distantly related groups evolved structures for temporary adhesion, among other convergent morphologies, independently [33,69,70]. Within free-living flatworms several characters are shared with respect to their adhesive organs [33,35,36,38]. First, adhesive organs comprise two gland cells, an adhesive gland, and a releasing gland whose necks penetrate a modified epidermal cell called the anchor cell. Second, the adhesive gland cell contains dense membrane-bound granules while the releasing gland has smaller vesicles with less-dense membrane bound granules. Third, adhesive gland cell necks are always surrounded by a microvilli collar either as an individual gland neck or in conjunction with the releasing gland neck.

The structural organization of adhesive organs within the order of the Macrostomida is quite stable [33,35,36]. Variation is e.g. limited to the number of microvilli of a collar, and the position of the gland- and anchor cell nuclei. Notably, in *Myozona sp*., for example, two adhesive cells and one releasing cell are present. In certain dolichomacrostomids the structural organization of the gland cell necks within the papillae are noteworthy: the adhesive gland cell neck in the papilla is folded into longitudinal ridges that exhibit a star-shaped morphology in an electron microscopical cross section. The releasing gland cell neck branches within the papilla and its finger-like extensions lie within the grooves of the adhesive cell folds [33,35]. The adhesive organs of *Macrostomum lignano*, and its close relatives [45]*Macrostomum tuba*[36] and *Psammomacrostomum sp.*[33] display comparable morphologies: an unbranched adhesive- and a releasing gland cell neck penetrate the anchor cell through a common pore. Together they are surrounded by a microvilli collar of a single anchor cell. For *M. lignano* we corroborated that 1) one adhesive organ is composed of exactly three cells, 2) the adhesive- and the releasing gland cell neck penetrate the anchor cell through a common pore, 3) 20–24 microvilli form the collar of the anchor cell 4) the microvilli possess a dense core, 5) the cytoplasm of anchor cells is enforced by intermediate filaments, and 6) hemidesmosomes connect the anchor cells to the extracellular matrix. Here, for the first time, we have provided evidence on the function of the anchor cell. By knocking-down essential structural elements, the intermediate filaments of the anchor cell, we could demonstrate that the structural integrity of this cell is critical for the adhesive function of *M. lignano*. Further investigations will be required to understand the molecular composition of the adhesive- and releasing gland cell secretions.

### The intermediate gene family

Intermediate filaments play an essential role in the cell integrity of many tissues and build a huge heterogeneous gene family [40-42]. For example the human genome encodes for approximately 70 different intermediate filaments and more than 30 diseases are related to mutations in these genes [43]. The members of this family have been divided into five groups, based on their gene- and primary structure: type I and II, keratins; type III, cytoplasmic IFs; type IV, neurofilaments; and type V, nuclear lamins [71]. IFs have been described as stress-absorbing elements and their main function is assumed to provide mechanical strength to cells [41,42]. In recent years additional functions beyond structural support have been identified. There is evidence that IFs contribute to the regulation of signalling pathways involved in cell survival, cell growth and cell polarity [40,72]. In contrast to the growing information on vertebrate IFs, little is known about invertebrate IFs. In this paper we describe a new cell-type specific, cytoplasmic IF in the platyhelminth *M. lignano*. The predicted protein Macif1 shares the common tripartite domain structure with a helical rod domain being flanked by nonhelical sequences at the head- and tail domain. Macif1 has the long, lamin-like length of the coil 1b domain, but lackes the specific motifs KRS/KR and CK/AIM conserved in lamins. The middle region of the coil 2 domain shows an irregularity in the heptad structure, the so-called stutter. These features seem characteristic for almost all invertebrate IFs described so far [67,73,74].

Using the amino acid sequence of Macif1 as the query, 11 additional sequences encoding for IFs could be found in the transcriptome database of *M. lignano* (http://www.macgenome.org) showing amino acid similarities from 23-88% in the conserved regions. So far there is no information available on their expression and function. We noted a staining of cells located around the mouth by *macif1* in situ hybridization (Figures 5A and 7A). This weak expression in the mouth is most likely due to a cross reaction with another intermediate filament variant. We have generated a short (204 bp) *in situ* probe from the 5’ region of *macif1* (28 bp – 231 bp). This probe showed exclusive expression in the anchor cells. However, because of the weaker staining of the short probe, we preferred to use the 558 bp long *in situ* probe for the experiments. Furthermore, we did not observe any effect in the mouth region or in the feeding behaviour in *macif1* RNAi treated animals. Macif1 shows a high similarity (74% amino acid identity) to the IF variant DjIFb of the platyhelminth *Dugesia japonica*. DjIFb is expressed specifically within the body margin, resembling the region of the adhesive organs in this animal [39]. The cell type expressing DjIFb could not be identified, but was described as located in the mesenchyme with projections into the epidermal layer. The description of the cellular shape and the location within the body margin coincides to the anchor cells of the respective adhesive organs. The expression pattern, together with the sequence similarity indicates Macif1 and DjIFb as homologues proteins with possible conserved function.

### Role of Macif1 during attachment

**Figure 8:**
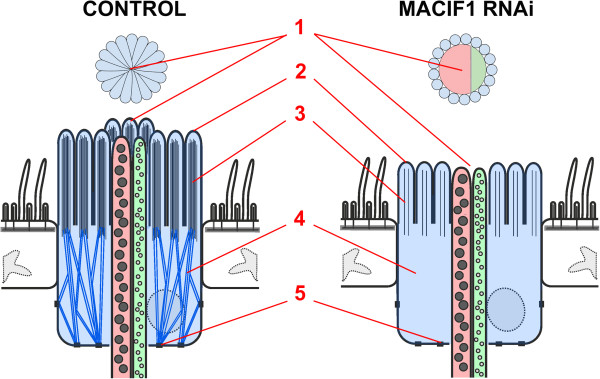
**Schematic illustration of an adhesive organ in controls and in macif1 dsRNA treated animals.** Anchor cell (blue), adhesive (red) and releasing gland (green) within the epidermis (white). Five morphological alterations are obvious. 1) The microvilli core of *macif1* dsRNA treated animals is closed in controls, whereas it appears open in treated animals. 2) The microvilli are shorter and 3) miss the dense core of actin filaments. 4) The treated animals lack intermediate filaments in the cytoplasm of anchor cells and 5) a reduced cytoplasmic interaction with hemidesmosomes is present.

The functional knock-down of Macif1 using RNA interference led to the first described non-adhesion phenotype of a marine organism. We showed that a cell-specific, cytoplasmic IF was crucial for the attachment of *M. lignano*. The lack of IFs within anchor cells led to severe morphological alterations in the respective cells. We observed five alterations upon *macif1* RNAi treatment (summarized in Figure 8): First, the tips of the microvilli of the papillae were open while normal papillae possessed a microvilli-enwrapped tip. Second, the microvilli were shorter and, third, the dense actin filament core of the microvilli was narrow or completely missing. Fourth, the cytoplasm of the anchor cell lacked IFs. Finally, the hemidesmosomes of *macif1* dsRNA treated animals possessed less electron-dense material at the cytoplasmic side compared to controls. The effect of IFs on microvilli structure and integrity has been shown before in human epithelial cell lines [75]. In that study the apically expressed IF cytokeratin 19 was down-regulated with specific antisense oligonucleotides. The treated cells exhibited a lower number of microvilli on their apical surface and a severe decrease of apically located F-actin. The study also suggested a correlation between the presence of IFs and villin, a protein that is known to support actin bundling in microvilli [75]. A similar phenotype has been found in intestinal cells of mice lacking the IF variant cytokeratin 8 [76]. In cytokeratin 8 knockout mice the villus enterocytes lack all cytoplasmic IFs. Ultrastructural analyses of these cells revealed microvilli with only 20% of their normal length. Moreover, the cells were devoid of several apical membrane proteins, suggesting that the apical polarization is disrupted in the absence of IFs [76]. In contrast, the functional knock-down of the intestine-specific intermediate filament IFC-2 in *Caenorhabditis elegans* resulted in bubble-shaped invaginations in the endotube, but did not affect the ultrastructure of microvilli, localization of actin filaments, or apical polarization of the cells [77]. However, IFC-2 represents just one of six intestinally-expressed IFs in *C. elegans* and a simultaneous down-regulation of two or three IFs led to a much more severe phenotype [78]. Therefore, it is possible that the respective IFs act in a redundant manner. In summary, a feedback loop seems to be present in flatworms, mice, and humans concerning the morphology of the microvilli. The amount of intermediate filaments in the epithelial cells, either experimentally induced or based on pathology, alters the morphology of the microvilli of the respective epithelium.

As the underlying mechanism of *M. lignano* attachment and detachment is not understood yet, one cannot define how the alterations found in *macif1* down-regulated animals disrupt the attachment process. It was hypothesized that microvilli are glued to the surface by the secretion material of the adhesive cell. Adhesive forces are then transmitted to the tail-plate by the filament-enforced anchor cell which is linked to the extracellular matrix by hemidesmosomes. Releasing gland secretion dissolves or depolymerizes the adhesive material and enables detachment [33]. We currently have no evidence about the nervous control of the secretory activity of the cells of the *M. lignano* adhesive organ. We showed that the morphological changes of the anchor cell induced by macif RNAi impeded adhesion. However, which of these changes (Figure 8) is responsible for the inability to perform adhesion cannot be discriminated. We have never observed any morphological evidence for a mechanosensory function for microvilli of the *M. lignano* adhesive organs. Interestingly, the effect of the *macif1* RNAi treatment was still visible after two weeks of recovery. The first worms to adhere again were identified 11 days after the end of the treatment with dsRNA. This suggests that the homeostasis of the anchor cells is a slow process compared to their fast regeneration ability.

## Conclusion

The ability to adhere to the substrate is manifested in all major phyla that are members of the interstitial meiofauna. Flatworms have evolved a duo-gland adhesive- and release system that performs efficiently in terrestrial, marine, and freshwater environments in the case of free-living flatworms and within the host tissues in the case of parasitic Platyhelminthes. We showed the detailed morphology of *Macrostomum lignano* adhesive organs. We assume that the microvilli of the anchor cells are glued to the surface and provide structural support during the adhesion process. Our results reveal that an intermediate filament variant is crucial for the attachment of a whole organism and that the structural integrity of the anchor cells is essential for the adhesive function. Experimental or clinical alterations of intermediate filaments in mice and humans also result in loss of mechanical support of cells. The rapid regeneration of the *M. lignano* adhesive organs after amputation and the available toolbox render this model organism as a suitable model system to study the function of intermediate filaments and to unravel the molecular foundation of flatworm adhesion.

## Material and methods

### Animal culture

*Macrostomum lignano*[48] cultures of the inbred line DV1 [79] were kept in petri dishes with nutrient enriched artificial seawater (Guillard's f/2 medium) [80] and were fed *ad libitum* with the diatom *Nitzschia curvilineata.* Animals were maintained in a climate chamber with 20°C, 60% humidity and a 14:10 day-night cycle.

### Electron microscopy

Chemical fixation and cryo-processing (high pressure freezing and freeze substitution) of DV1 *M. lignano* for transmission electron microscopy (TEM) and scanning electron microscopy (SEM) were performed as described in previous studies [51,59]. High pressure freezing was performed using a HPM-010 (HPF apparatus from BAL-TEC, Baltzers, Liechtenstein). Specimens were stored in liquid nitrogen and freeze-substituted in acetone (containing 0.5% osmium tetroxide and 0.1% uranyl acetate), embedded in Polybed 812 and cut with a Leica ultramicrotome UCT (Leica, Vienna). Sections were stained with lead citrate and examined with a Zeiss Libra 120 TEM (Zeiss, Germany).

RNAi treated specimens and their controls were fixed with 2.5% glutaraldehyde in 0.1 M cacodylate buffer, postfixed with 1% osmium tetroxide. Samples for TEM were dehydrated in an acetone series embedded in Polybed 812, cut and double stained with uranyl acetate and lead citrate, and examined with a Zeiss Libra 120 TEM (Zeiss, Germany). Images were made using the Olympus SiS iTEM 5.0 software and a TRS 2048 high speed camera. For SEM a mixture of 4% glutaraldehyde and 0.05% osmium tetroxide as a cocktail was used for a short pre-fixation, followed by subsequent chemical fixation with 4% glutaraldehyde and 1% osmium tetroxide in cacodylate buffer. SEM samples were dehydrated in a series of methanol, critical point dried, sputtered with gold, and examined with a Zeiss DSM 950 and a Zeiss DSM 982 Gemini (Zeiss, Germany).

### Gene isolation and primers

The intermediate filament *macif1* was identified during an *in situ* hybridization screen [65] of a tail-specific subset of transcripts identified using a positional RNA-Seq screen [66]. The gene is 99% identical (1531 out of 1543 bp) to the Contig1120 available in the publically accessible database http://flatworm.uibk.ac.at/macest/. We isolated the gene using the primer pairs 5′-ATGGCTAGCAAGACAACCACC −3′ and 5′-ATTTTCTTGAACTGTTTCAATAGATGG-3′. The obtained fragment was cloned into pGEM-T (Promega) and sequenced by Microsynth (Switzerland). The full-length sequence comprises 1815 bp. The gene sequence of *macif1* was submitted to GenBank [GenBank: KF441715].

### Whole mount *in situ* hybridization

Whole mount *in situ* hybridization (ISH) was modified after [81] with the following changes: (1) Template DNA for producing DIG-labelled *macIF* probe (558 bp) was made using Phusion® polymerase (New England Biolabs) with the primer couple 5′-AAGGAGACTGAGCGAGTGAAGC-3′ and 5′-GGATCCTAATACGACTCACTATAGGCATGACGTCCATCTTGTTGTCG-3′. (2) After hybridization, animals were transferred from the reaction tubes into 24 mesh baskets (53 μm mesh size; INTAVIS Bioanalytical Instruments AG, Germany) which were placed into custom made holes drilled into the lid of a 24-well tissue culture plate. Plates with pre-warmed buffers were prepared and the lid with the baskets and animals was transferred to the successive solution. For colour development animals were moved to a plate without baskets.

For semi-thin sections whole mount *in situ* hybridizations were slightly overstained and fixed in BOUIN’s fluid for several hours. Specimens were dehydrated in an ethanol series and embedded in PolyBed 812 and polymerized for 48 hours. Specimens were cut serially with 2 μm semi-thin sections using a Reichert Autocut (Reichert, Vienna) and a Diatome Histobutler diamond knife (Diatome, Switzerland). Sections were examined with a Leica DM5000B microscope (Leica, Germany) microscope, a Leica DFC490 digital camera and Leica application suite software.

### Phalloidin labelling

Animals were relaxed with 7.14% MgCl_2_ hexahydrate and then fixed in 4% formaldehyde (made from paraformaldehyde) in PBS for 30 min (pH 7.4), washed 3 times 15 min with PBS-Triton 0.1% and then incubated in Alexa 488 phalloidin (1/300) (Invitrogen) for 1 hour at RT in the dark. They were then washed again three times in PBS-Triton for 10 min. Specimens were mounted in Vectashield and analysed using a Leica DM5000 or a Leica SP5 II confocal scanning microscope. For super resolution microscopy the Alexa 488 phalloidin stained specimen were mounted in Mowiol and examined with a Leica TCS SP8 gSTED microscope system.

### RNA interference

RNAi was performed as previously described [51]. Briefly, double-stranded RNA (dsRNA) probe was generated by an *in vitro* transcription system (T7 Ribomax™ large scale RNA kit, Promega) which overlapped in sequence completely with the ISH probe (bp366–bp921). 250 μl of dsRNA was applied to the medium to a final concentration of 15 ng/μl. In order to eliminate Macif1 protein completely, we amputated the tail plate and treated the animals with *macif1* dsRNA during the entire regeneration process. 25 animals were kept in each well of a 24-well plate. Supernatant was changed every 24 h. Throughout the whole experiment, animals were fed *ad libitum*. As a negative control f/2 culture medium was used. We showed in earlier studies that control animals treated with dsRNA of a nonendogenous gene did not show any mock effect [50,52,53]. Therefore, in the present study, control animals were only kept with 250 μl f/2 culture medium. At different time points *macif1* dsRNA treated animals and controls were individually transferred to a slide and observed for one minute to score the number of successful attachments to the slide.

## Abbreviations

dsRNA: double-stranded RNA; IF: Intermediate filament; RNAi: RNA interference; SEM: Scanning electron microscopy; STED: Stimulated emission depletion; TEM: Transmission electron microscopy.

## Competing interests

The authors declare that they have no competing interests.

## Authors’ contributions

PL and BL conceived and designed the study, performed experiments, and wrote the paper. RP, JW, MR, performed experiments and revised the paper. RA, LS, EB provided currently unpublished data that facilitated gene isolation. BE, MH, KP, SO, WS performed electron microscopy experiments and WS contributed to writing the paper. All authors read and approved the final manuscript.

## Supplementary Material

Additional file 1 **Cross section of the tail plate at the posterior end of the horse-shoe shaped adhesive system (cryo-processed specimen).** Dorsal is to the top. About 95 adhesive organs are visible on this section. On the left half cells are false-colour coded for clarity: anchor cells (blue), adhesive gland cell necks (red), releasing gland cell necks (green). Note that almost all adhesive gland cell necks are located at the ventral side within the adhesive organs. Scale bar 5 μm. Click here for file

Additional file 2 **Ultrastructure of an adhesive gland cell (A) and releasing gland cell (B) and distribution of the cell types in the overlapping region (cryo-processed specimens) (C).** Inset **(A)** developing adhesive granules in trans golgi region (asterisk). adg adhesive granules; bwm body wall musculature; ep epidermis; er endoplasmic reticulum; go golgi apparatus; mi mitochondrium; muc mucus gland; nu nucleus; nc nerve cord; rg releasing granules; stg storage granules. I-VI adhesive gland cell bodies; 1–4 releasing gland cell bodies. Scale bars **(A, B)** 1 μm, **(C)** 5 μm. Click here for file

Additional file 3 Video of control animals after 9 days of regeneration. Click here for file

Additional file 4 **Video of *macif1 *** **RNAi treated animals after 9 days of regeneration.** Click here for file

Additional file 5 **Recovery of adhesion.** Animals were tail-amputated at day 0 and left regenerating in normal culture medium (controls) or treated with *macif1* dsRNA. At day 13 post-amputation animals were transferred of from *macif1* dsRNA treatment to normal culture medium (i.e. recovery animals) while control animals were kept on normal culture medium all time. Note that recovery animals started to adhere only at 11 days after transfer to normal culture medium. Click here for file

Additional file 6 **Comparison of controls and *macif1* dsRNA treated animals. (A-B)** Overview and detail of a whole mount *in situ* hybridization of *macif1* in a control **(A)** and *macif1* dsRNA treated animal **(B)** at day 9 post amputation. **(C, D)** Phalloidin labeling at day six post-amputation. **(C)** Control animals regenerated normal adhesive organs (arrowheads) while *macif1* dsRNA treated animals **(D)** exhibited shorter organs. Note that **(D)** required a higher gain in confocal microscopy to visualize the adhesive organs. bmw body wall musculature; stm stylet muscles. Scale bars **(A, B)** 100 μm, **(A’, B’)** 20 μm, **(C, D)** 30 μm. Click here for file
