# Supplementary figures and images for: Biological adhesion of the flatworm Macrostomum lignano relies on a duo-gland system and is mediated by a cell type-specific intermediate filament protein
Source: Front Zool. 2014 Feb 12;11:12. doi: 10.1186/1742-9994-11-12 (PMC4016567; doi:10.1186/1742-9994-11-12)

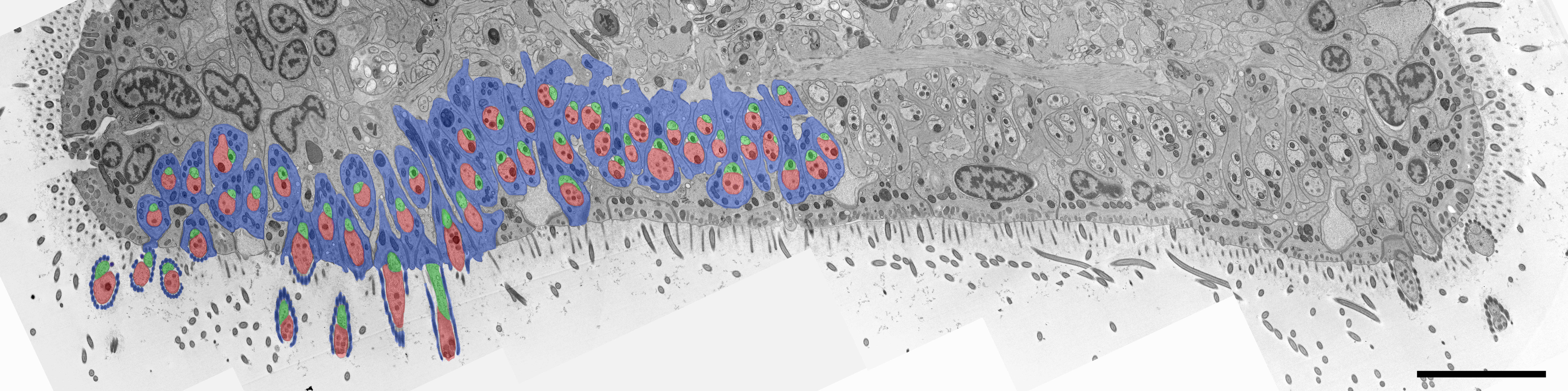

Supplement: Additional file 1 — Cross section of the tail plate at the posterior end of the horse-shoe shaped adhesive system (cryo-processed specimen). Dorsal is to the top. About 95 adhesive organs are visible on this section. On the left half cells are false-colour coded for clarity: anchor cells (blue), adhesive gland cell necks (red), releasing gland cell necks (green). Note that almost all adhesive gland cell necks are located at the ventral side within the adhesive organs. Scale bar 5 μm. [file 1742-9994-11-12-S1.jpeg]

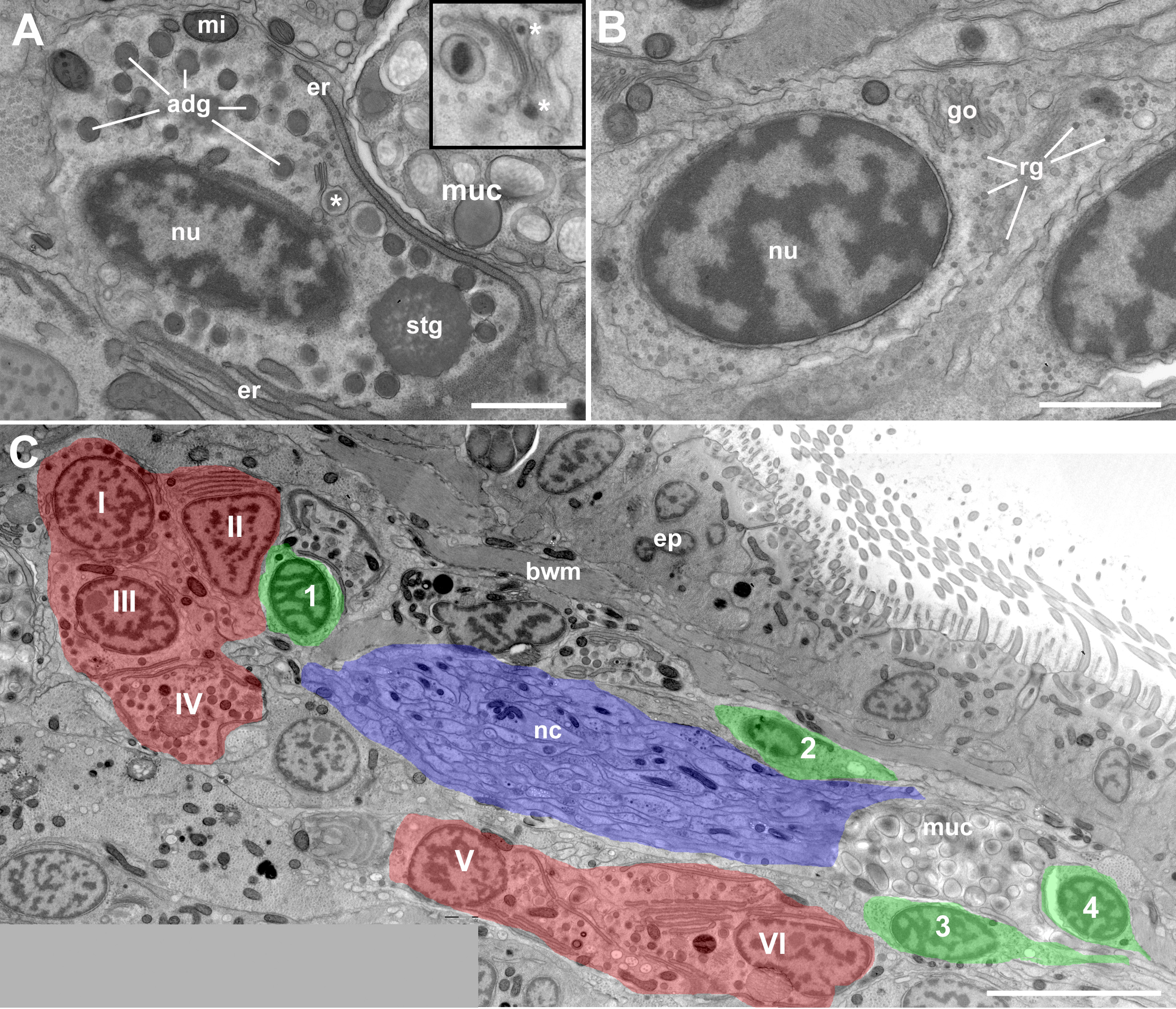

Supplement: Additional file 2 — Ultrastructure of an adhesive gland cell (A) and releasing gland cell (B) and distribution of the cell types in the overlapping region (cryo-processed specimens) (C). Inset (A) developing adhesive granules in trans golgi region (asterisk). adg adhesive granules; bwm body wall musculature; ep epidermis; er endoplasmic reticulum; go golgi apparatus; mi mitochondrium; muc mucus gland; nu nucleus; nc nerve cord; rg releasing granules; stg storage granules. I-VI adhesive gland cell bodies; 1–4 releasing gland cell bodies. Scale bars (A, B) 1 μm, (C) 5 μm. [file 1742-9994-11-12-S2.jpeg]

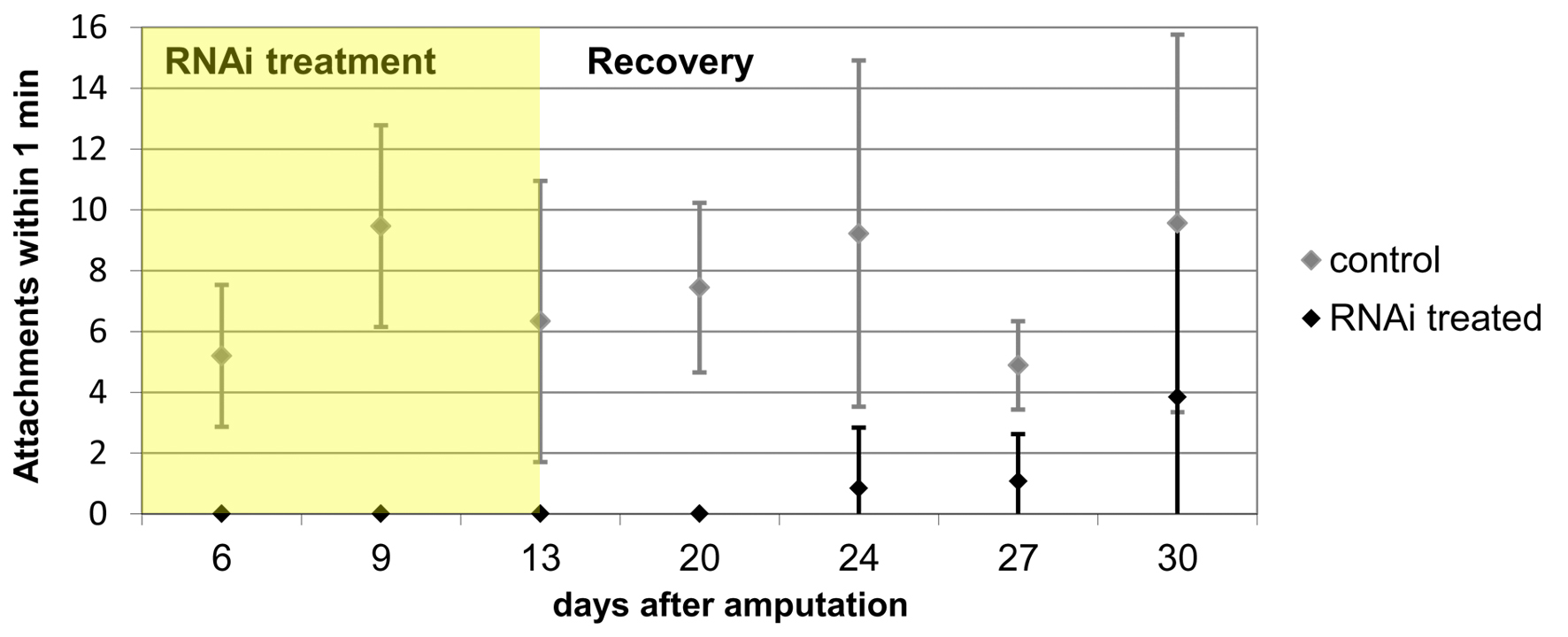

Supplement: Additional file 5 — Recovery of adhesion. Animals were tail-amputated at day 0 and left regenerating in normal culture medium (controls) or treated with macif1 dsRNA. At day 13 post-amputation animals were transferred of from macif1 dsRNA treatment to normal culture medium (i.e. recovery animals) while control animals were kept on normal culture medium all time. Note that recovery animals started to adhere only at 11 days after transfer to normal culture medium. [file 1742-9994-11-12-S5.jpeg]
